# Acute liver effects, disposition and metabolic fate of [^14^C]-fenclozic acid following oral administration to normal and bile-cannulated male C57BL/6J mice

**DOI:** 10.1007/s00204-016-1894-5

**Published:** 2016-11-28

**Authors:** Kathryn Pickup, Scott Martin, Elizabeth A. Partridge, Huw B. Jones, Jonathan Wills, Tim Schulz-Utermoehl, Alan McCarthy, Alison Rodrigues, Chris Page, Kerry Ratcliffe, Sunil Sarda, Ian D. Wilson

**Affiliations:** 1 Drug Metabolism and Pharmacokinetics, Oncology iMED Chesterford Science Park, AstraZeneca UK Ltd., Saffron Walden, Essex, CB10 1XL UK; 2 Discovery Sciences IM, Milton Science Park, AstraZeneca UK Ltd., Cambridge, Cambridgeshire, CB40FZ UK; 3 Drug Metabolism and Pharmacokinetics IM, AstraZeneca UK Ltd, Alderley Park, Macclesfield, Cheshire, SK10 4TG UK; 4 Global Safety Assessment Department, Alderley Park, AstraZeneca UK Ltd, Macclesfield, Cheshire, SK10 4TG UK; 5 In Vivo Assays Ltd, c/o Biohub, Alderley Park, Macclesfield, SK10 4TG UK; 6 Molecular and Clinical Pharmacology, MRC Centre for Drug Safety Science, Sherrington Building, University of Liverpool, Ashton Street, L69 3GE Liverpool, UK; 7 Cyprotex Discovery Ltd, 15 Beech Lane, Macclesfield, Cheshire, SK10 2DR UK; 8 Agilent Technologies Inc., 5500 Lakeside, Cheadle, SK8 3GR UK; 9 Sygnature Discovery, DMPK Department, BioCity, Nottingham, NG1 1GF UK; 10 Department of Surgery and Cancer, Imperial College, Exhibition Rd, South Kensington, London, SW7 2AZ UK

**Keywords:** Fenclozic acid, Mouse, Reactive metabolites

## Abstract

The distribution, metabolism, excretion and hepatic effects of the human hepatotoxin fenclozic acid were investigated following single oral doses of 10 mg/kg to normal and bile duct-cannulated male C57BL/6J mice. Whole body autoradiography showed distribution into all tissues except the brain, with radioactivity still detectable in blood, kidney and liver at 72 h post-dose. Mice dosed with [^14^C]-fenclozic acid showed acute centrilobular hepatocellular necrosis, but no other regions of the liver were affected. The majority of the [^14^C]-fenclozic acid-related material recovered was found in the urine/aqueous cage wash, (49%) whilst a smaller portion (13%) was eliminated via the faeces. Metabolic profiles for urine, bile and faecal extracts, obtained using liquid chromatography and a combination of mass spectrometric and radioactivity detection, revealed extensive metabolism of fenclozic acid in mice that involved biotransformations via both oxidation and conjugation. These profiling studies also revealed the presence of glutathione-derived metabolites providing evidence for the production of reactive species by mice administered fenclozic acid. Covalent binding to proteins from liver, kidney and plasma was also demonstrated, although this binding was relatively low (less than 50 pmol eq./mg protein).

**Electronic supplementary material:**

The online version of this article (doi:10.1007/s00204-016-1894-5) contains supplementary material, which is available to authorized users.

## Introduction

Fenclozic acid (2-(4-chlorophenyl)-thiazol-4-ylacetic acid) (Myalex) was identified as a promising anti-rheumatic in the 1960’s that exhibited no adverse hepatic effects in pre-clinical animal tests or initial studies in man (Chalmers et al. 1969a, b). However, whilst doses of 100 mg twice daily were well tolerated, doses of 400 mg resulted in a relatively high incidence of jaundice (2 out of 12 subjects in one centre) (Hart et al. 1970). As further cases of jaundice and abnormal hepatic function were detected in other trials where the 400 mg dose of fenclozic acid was administered clinical studies were halted and development was terminated (Alcock 1970). Following the termination of development, the pre-clinical data for fenclozic acid were re-examined and additional work, in a wider range of pre-clinical species, was undertaken in an effort to understand the toxicity of the compound but to no effect (Alcock 1970). Fenclozic acid therefore stands as an example of a drug candidate with a good pre-clinical safety profile and good efficacy that nevertheless caused serious, dose-related, non-idiosyncratic adverse drug reactions (ADR’s) in man. Whilst such examples of human-specific ADRs are fortunately rare, they raise questions regarding the effectiveness of pre-clinical safety studies that would benefit from a thorough mechanistic investigation of the cause(s) of the toxicity. Recent studies showed that the bioactivation of fenclozic acid in vitro resulted in high levels of covalent binding to microsomal preparations supplemented with NADPH (Rodrigues et al. 2013), but not UDPGA. So, whilst the reactive species responsible for the covalent binding proved refractory to identification, in either microsomal or hepatocyte-based systems, it seemed likely that oxidative metabolism was responsible for the bioactivation of the drug. Indeed, although potentially reactive acyl glucuronides are also formed during the metabolism of fenclozic acid in vitro covalent binding in microsomal systems supplemented with UDPGA was lower than systems optimised for oxidative metabolism. Further studies in the HRN™ (hepatic reductase null) mouse, where hepatic CYP450s are inactive and the drug is essentially subject to biotransformation via acyl conjugation to glucuronic acid and amino acid conjugates, showed only low levels of hepatic covalent binding (Pickup et al. 2014). More recently, studies in bile duct-cannulated rats showed the presence of metabolites that were clearly derived from reactive metabolites detoxified via reaction with glutathione (Martin et al. 2014). However, as this study did not employ radiolabelled material, it was not possible to determine the contribution of this route to the overall metabolic fate of the drug. Here, the metabolic fate and hepatic effects of [^14^C]-fenclozic acid in normal and bile duct-cannulated male C57BL/6J mice have been investigated.

## Experimental procedures

### Chemicals

Unlabelled fenclozic acid (2-(2-(4-chlorophenyl)thiazol-4-yl)acetic acid) was obtained from Compound Management (AstraZeneca, Alderley Park, Cheshire, UK), batch number: AZ10002189-024. [^14^C]-fenclozic acid (2-(2-(4-chlorophenyl)[2-^14^C]thiazol-4-yl)acetic acid) was synthesised and supplied by Isotope Chemistry, AstraZeneca, at 237 μCi/mg and a radiochemical purity >99%. Ultima Gold scintillation cocktail was purchased from Packard Instruments, (Pangbourne, UK). CarboSorb oxidiser absorbant and Permafluor E+ scintillant were obtained from PerkinElmer (Beaconsfield, UK), whilst FLUOTHANE™ was obtained from the AstraZeneca Group of Companies. Pierce BCA protein assay kit was obtained from Perbio Science (Cramlington, UK). Acetonitrile, ethanol, formic acid, hexane, tetrahydrofuran (THF), trifluoroacetic acid (TFA) and xylene were all of analytical grade and supplied by Fisher Scientific (Loughborough, UK). Phosphate buffer (pH 7.4) was supplied by AZ media (AstraZeneca). All other chemicals or solvents were purchased from commercial suppliers and were of analytical grade or the best equivalent.

### Animals

Fifteen wild-type mice (strain: C57BL/6J) aged approximately 8 weeks and weighing between 22.8 and 27.2 g were supplied by the AstraZeneca Rodent Breeding Unit, Alderley Park. All animal procedures and treatments were carried out in accordance with approved animal licenses and guidelines issued by the British Home office (Animals (Scientific Procedures) Act (1986)).

## Study design

All of the mice used in these studies were identified by unique tail markings, and acclimatised for 3 days before dosing. Mice were kept at room temperature and exposed to a 12 h dark/12 h artificial light cycle and an R&M No. 1 Modified Irradiated diet; potable water was available ad libitum. Mice were fasted overnight for 12 h prior to oral administration (by gavage) of either [^14^C]-fenclozic acid (10 mg/kg) or phosphate buffer (dosing volume of 5 mL/kg) the following morning. Phosphate buffer (pH 7.4) was used as the vehicle control dose.

*Study 1* Semi-quantitative whole body autoradiography (QWBA) was performed in order to determine the distribution of [^14^C]-fenclozic acid (dose 10 mg/kg and 200 μCi/kg) following oral administration to 6 male C57BL/6J mice. [^14^C]-Fenclozic acid, specific activity of 237 μCi/mg, was diluted with unlabelled fenclozic acid to give material with a specific activity of ca. 110 μCi/mg. Unlabelled fenclozic acid was dissolved in phosphate buffer (pH 7.4) and an appropriate volume of this solution was added to the [^14^C]-fenclozic acid to give a solution at the target-specific activity 20 μCi/mg and a target concentration of 2 mg/mL. After dosing, the mice were housed individually in polycarbonate cages with stainless steel mesh inserts, with two animals killed at each of the following time points, 6, 24 and 72 h post-dose by FLUOTHANE™ inhalation. Carcasses were immediately frozen in a mixture of hexane and dry ice at ca. −80 °C. Following freezing, the hind legs and tail were removed and each carcass was then embedded (left side uppermost) in a block of carboxymethylcellulose (ca. 2% in water). The blocks were mounted on the stage of a Leica CM3600 cryomicrotome (Leica Microsystems, Germany) maintained at approximately −20 °C. Sagittal sections (30 µm) were prepared from each animal based on the work of Ullberg (Ullberg 1954) to include as many tissues as possible. Sections were then allowed to freeze-dry prior to exposure to FUJI phosphor imaging plates (Raytek, Sheffield UK). Sections for imaging were enclosed in a light-tight cassette and were exposed for 7 days. After exposure, the imaging plates were removed from the cassettes under dark room conditions and the plates scanned using a FUJI FLA 5000 phosphor imager. Images were obtained and quantified using AIDA version 4.22 (Raytest UK, Chesterfield UK).

*Study 2* A study was undertaken to profile the metabolites present in the excreta of male C57BL/6J mice (*n* = 3) [^14^C]-fenclozic acid following oral administration (dose 10 mg/kg and 1100, μCi/kg in pH 7.4 phosphate buffer, with a dosing volume of 5 mL/kg). [^14^C]-Fenclozic acid, specific activity 237 μCi/mg, was diluted with unlabelled fenclozic acid to give material with a specific activity of ca. 110 μCi/mg as described above. A further 3 mice were administered phosphate buffer to provide a vehicle only control dose for histopathology (see below). Mice were housed individually in glass metabolism cages and urine, faeces and aqueous cage wash were collected from individual animals for 72 h post-dose (urine and cage wash 0–6, 6–12 and 12–24 h then every 24–72 h, faeces were collected 0–24, 24–48 and 48–72 h, and samples were collected over dry ice to ensure sample stability), after which animals were killed by FLUOTHANE™ inhalation. Following death, the mice were exsanguinated and the blood spun for 2 min, at approximately 7800 g and ambient temperature to produce plasma. The liver (with gall bladder removed) and kidneys from each animal were removed, a small sample of each liver was taken and fixed for histopathology, and the remainder of the liver and kidneys were snap-frozen in liquid nitrogen.

*Study 3* A third study was undertaken to obtain metabolite profiles for [^14^C]-fenclozic acid in mouse bile following oral administration of 10 mg/kg (200 μCi/kg) to bile duct-cannulated C57BL/6J mice. [^14^C]-Fenclozic acid, specific activity of 237 μCi/mg, was diluted with unlabelled fenclozic acid to give material with a specific activity of ca. 110 μCi/mg. Unlabelled fenclozic was dissolved in phosphate buffer (pH 7.4), and an appropriate volume of this solution was added to the [^14^C]-fenclozic acid to give a solution at the target-specific activity 20 μCi/mg and a target concentration of 2 mg/mL. The bile duct-cannulation procedure has been described in detail elsewhere (Sarda et al. 2014), briefly, within 20 min of dosing 5 animals, plus an undosed control mouse, were anaesthetised and, following a midline incision (*linea alba*) the bile duct was cannulated and the cannula was exteriorised, with the bile then collected into a 0.5 mL Eppendorf tube adjacent to the animal. Mice were maintained under anaesthesia until 6 h post-dose at which point animals were killed by halothane inhalation. As much urine as possible was sampled from the bladder at 6 h post-dose, but no attempt was made to make a complete collection. Blood was also collected (into heparin) and plasma prepared. Samples were frozen on collection and stored at −20 °C until analysis.

### Histopathology

Liver samples from mice from Study 2 were fixed in 10% neutral-buffered formaldehyde for 5 days before dehydrating in increasing concentrations of industrial methylated spirits (IMS), IMS and xylene (70, 80 and 95%) then xylene alone, over a total of 7 h. Dehydrated samples were embedded in paraffin wax, after which sections (3 μm thick) were prepared and stained with haematoxylin and eosin (H&E). Stained sections were observed using light microscopy.

### Determination of radioactivity in samples

Radioactivity in weighed aliquots of samples of urine, bile, aqueous cage wash and plasma was diluted to 1 mL with deionised water and mixed with 10 mL of scintillation cocktail (Ultima Gold). Radioactivity was determined by liquid scintillation counting (LSC) using a Packard TriCarb scintillation counter (Pangbourne, UK) for 10 min or until 10^6^ counts had accumulated. Samples of faeces, liver and kidney were first homogenised in water (1:3 w/v) and then combusted using a Packard Oxidiser (model 307). The products of combustion were trapped in 9 mL of CarboSorb, and then 12 mL Permafluor E+ scintillation cocktail were added prior to LSC as described above.

### Covalent binding

Liver and kidney samples from each animal from Study 2 were homogenised by tissue type in water, and individual plasma samples were used untreated. The radioactive content for each sample was measured by scintillation counting. The protein content of the homogenates and plasma was determined using a Pierce BCA protein assay kit, and samples diluted to give 2 mg/mL protein using 0.1 M phosphate buffer (pH 7.4). Aliquots (200 µL) were then placed in the wells of a 96-well plate and 300 µL of acetone added, with the samples then agitated for 1 min. A further 500 µL acetone was then added and the samples agitated again for 1 min to allow the protein to precipitate. The protein pellet was washed with 80% methanol in water using a Brandel cell harvester (Gaithersburg, MD). The protein concentration and radioactive content of the remaining pellet were determined by scintillation counting and the extent of covalent binding calculated by difference. The statistical significance of the difference was calculated using the Student’s *t-*test function in Microsoft Excel.

## Sample preparation for [^14^C]-metabolite profiling and identification

Urine, faeces, plasma and bile samples were pooled by excreta type to include any sample containing greater than 1% of the administered dose. The radioactive content of each pool was determined either by LSC directly (urine, and bile) or following combustion (faecal homogenates) as described above. Pre-dose urine and faeces samples were also prepared in the same way as the post-dose samples.

Pooled urine samples were concentrated to ca. 400,000 dpm/mL under a stream of oxygen-free nitrogen. Bile samples were pooled to produce a single 0–6 h post-dose sample and centrifuged at ca. 12,000*g* for 5 min prior to analysis. The supernatants were transferred to an equal volume of ultra-purified distilled water in Waters HPLC 2 mL vials for LC/MS analyses. The 6-h plasma samples were pooled and a 100 µL aliquot taken and mixed with 300 µL of acetonitrile to precipitate proteins, followed by centrifugation at 12,000*g* for 5 min. The supernatants were transferred to an equal volume of ultra-purified distilled water in Waters HPLC 2 mL vials to produce a sample for analysis.

The aqueous faecal homogenates were first extracted with acetonitrile which was added to each homogenate in the ratio 3:1 (vol/weight) and the sample mixed on a rotary mixer for 10 min before centrifuging at 3345*g* for 10 min at ambient temperature. The supernatant was removed and the radioactive recovery determined by LSC. As only 78% of the total radioactivity was extracted from the faecal homogenate a further extraction was performed, first with 2% (v/v) formic acid in water (3:1vol/wt) followed by extraction with tetrahydrofuran (THF) (3:1vol/wt). These three extracts gave a combined recovery of 90% of the radioactivity present in the sample. Extracts were combined and then concentrated by rotary evaporation at 30 °C, followed by further concentration under a stream of oxygen-free nitrogen to give samples containing ca. 400,000 dpm/mL. The concentrated samples were centrifuged at 25,000*g* for 3 min to remove any particulate matter.

## Metabolite identification

### Structural characterisation of metabolites and metabolite profiling by UHPLC-LTQ-Orbitrap and TopCount radioactivity detection

For the analysis of urine, bile and faeces, a UHPLC-Orbitrap profiling system was used similar to that described by Martin et al. (2014). Accurate mass structural characterisation was performed using an LTQ Orbitrap XL mass spectrometer (ThermoFisher Scientific, Bremen, Germany) fitted with an electrospray source which was operated in either positive or negative ion mode. Chromatographic separation was via a Waters Acquity Ultra high-Performance Liquid Chromatography (UPLC) system. The modular Waters Acquity system consisted of a binary UPLC pump, column oven and autoinjector equipped with a photodiode array (PDA) detector with chromatography undertaken in a column oven at 50 °C on a Waters BEH C18 column (2.1 × 100 mm, 1.8 µm), preceded by a guard filter. Mobile phase consisted of 0.1% (v/v) of formic acid in water (eluent A) and 0.1% (v/v) formic acid in methanol (eluent B) and was delivered as a gradient at 0.45 mL/min. The gradient elution profile comprised an initial hold at 5% B for 1 min which was stepped to 20% B at 1.01 min, and a linear gradient applied to reach 50% B after 15 min, with a further increase to 98% B at 16 min, at which point it was held until 17 min, after which the column was re-equilibrated to 5% B for 2 min, making a total run time of 19 min. Samples were injected (20 μL) onto the column, and the eluent was diverted to waste for the first 1 min. In line UV spectra were acquired over 190–330 nm wavelength range. The eluent was split post-column 5:1 (v/v), to an HTC-PAL fraction-collector (Presearch Ltd., Basingstoke, UK) for collection into 96 deep-well Luma-plates™ coated with solid scintillant (Perkin Elmer Ltd., Seer Green, UK) across the whole acquisition at a rate of 2.4 s per well. The plates were then dried and analysed for radioactivity on a Packard HTS-TopCount-NXT (Packard Instruments, Pangbourne, UK). Full scan MS data were obtained over the mass range of 100 to 1200 Da. Targeted MS^2^ experiments were conducted in the Orbitrap using higher energy collisional dissociation (HCD) fragmentation, isolation width 3 Da, normalised collision energy 60 eV, and activation time 30 ms. All ion trap MS^n^ experiments were performed using collision induced dissociation (CID), isolation width 3 Da, normalised collision energy 35 eV, and activation time 30 ms. All ions acquired in the Orbitrap were monitored at 7500 resolution fwhm (full width at half-maximum).

### Data analysis

MS^n^ data were collected using Xcalibur v2.1 (ThermoFisher Scientific, Bremen, Germany) and TopCount radio-scintillation counts were reconstructed as radiochromatograms following import into LAURA v4.1.12.89 (LabLogic Systems Ltd., Sheffield, UK). Components were identified as being derived from fenclozic acid by interpretation of a combination of available datasets including characteristic isotopic patterns (mono-chlorinated), common fragments, UV absorbance, ^14^C incorporation and/or accurate mass. Comparisons with pre-dose control samples spiked with fenclozic acid were conducted to minimise the potential for false positives from system impurities and filter for endogenous components. All accurate mass measurements including the MS^2^ fragmentations were within ±3 ppm of the theoretical accurate mass.

## Results

### Histopathology

**Fig. 1:**
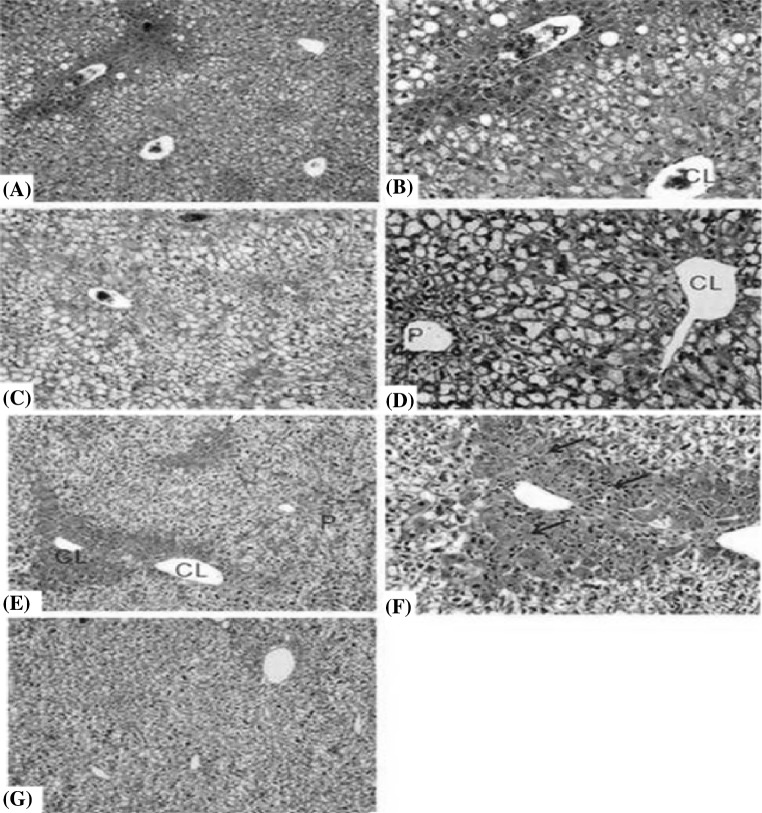
Photomicrographs of representative samples of the livers from wild type (WT) and hepatic cytochrome P450 reductase null (HRN) mice treated with a single 10 mg/kg oral dose of [^14^C] fenclozic acid (see Pickup et al. 2014). **a**, **b** HRN mice dosed with vehicle control, **c**, **d** HRN mice dosed with [^14^C]-fenclozic acid, and **e**, **g** WT mice dosed with [^14^C]-fenclozic acid. The images illustrate the absence of drug-induced toxicity in HRN mice in comparison with WT mice. **e**, **f** Representative of the pathology seen in WT mice following administration of [^14^C]-fenclozic acid with prominent centrilobular necrosis and little inflammatory cell infiltration (indicated by the *arrows*). **g** Taken from an animal also given [^14^C]-fenclozic acid but which showed no toxicity; this illustrates the variation in toxicity observed. Original objective lens magnification for **a**, **c**, **e**, **g** ×10 whereas **b**, **d**, **f** ×20. *CL* centrilobular and *P* periportal. Livers exposed to haematoxylin and eosin stains

Liver sections treated with haematoxylin and eosin stains observed under light microscopy are shown in Fig. 1. Two of the three mice dosed with [^14^C]-fenclozic acid showed acute centrilobular hepatocellular necrosis (indicated by the arrows in Fig. 1); no other regions were affected. The degree of inflammatory cell infiltration was minimal and related to the regions of centrilobular necrosis alone. Interestingly, in a previous study when fenclozic acid was administered to HRN mice at 10 mg/kg (as described in Pickup et al. 2014), where hepatic cytochrome P450s are non-functional, and there was no evidence of centrilobular necrosis, or indeed any other effect on the liver (see Fig. 1).

### Semi-quantitative whole body autoradiography (QWBA)

**Fig. 2:**
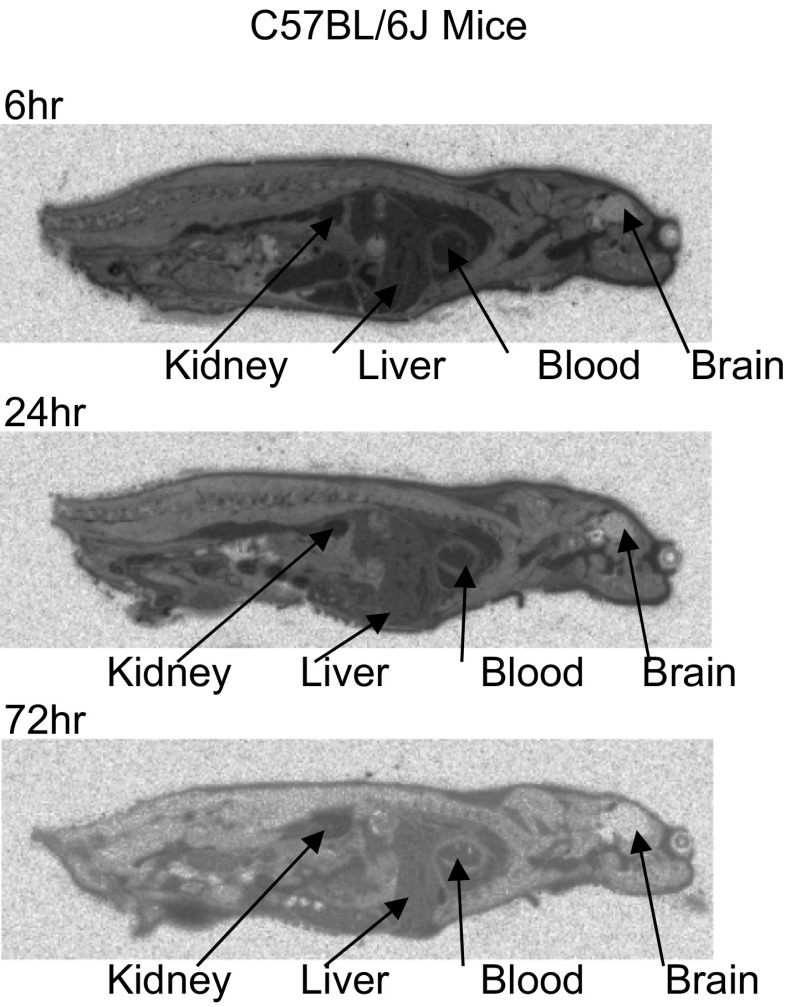
Autoradiography images of C57BL/6J mice following a single 10 mg/kg oral dose of [^14^C]-fenclozic acid. Fenclozic acid-related material was well distributed into all tissues with the exception of the central nervous system and still remained in tissues at 72 h post-dose

Following oral administration of [^14^C]-fenclozic acid QWBA showed that radioactivity was widely distributed into all the tissues of the mouse, with the exception of the CNS where only very low levels were detected (Fig. 2). The amounts of radioactivity determined from the autoradiographs for selected tissues are presented in Table 1, together with tissue/blood ratios, illustrating the retention of the radioactivity in the tissues compared with circulating drug-related material. The highest concentrations of radioactivity were detected in the blood, liver and kidney. The results of the QWBA study clearly showed that radioactivity was still detectable in the tissues of animals 72 h post-dosing.

**Table 1:** Radioactivity in liver, kidney and blood/tissue ratios via QWBA

WT	6 h		24 h		72 h	
	dpm/g	Tissue/blood	dpm/g	Tissue/blood	dpm/g	Tissue/blood
Blood	3150,670	1.00	1266,494	1.00	145,195	1.00
Brain	81,217	0.03	27,020	0.02	2143	0.01
Kidney cortex	3429,797	1.09	1565,700	1.24	298,993	2.06
Kidney medulla	1283,496	0.41	494,144	0.39	86,467	0.60
Liver	2187,659	0.69	626,466	0.49	123,707	0.85

The mean (*n* = 2) radioactive content (dpm/g) of selected tissues in of C57BL/6J mice, 6, 24 and 72 h after a single 10 mg/kg oral dose of [^14^C]-fenclozic acid

## Covalent binding of radioactivity to protein

The retention of radioactivity in the liver and kidneys of these mice as determined by QWBA was similar, with 0.63 ± 0.10 (SD) % of that administered remaining in the liver and 0.55 ± 0.10 (SD) % in the kidneys at 72 h post-dose. Further studies were therefore performed on plasma, liver and kidney samples to determine whether this relatively long-retained radioactivity was due to covalent binding (CVB) to cellular macromolecules. These studies showed CVB amounting to ca. 18, 40 and 10 pg/mg protein detected for liver, kidney and plasma, respectively (Table 2), which was slightly higher than that observed for these tissues in HRN mice given an equivalent dose in our previous study (ca. 13, 26 and 3 pg/mg protein for liver, kidney and plasma, respectively) (Pickup et al. 2014). Interestingly, these quantities of CVB were approximately an order of magnitude greater than those observed for the idiosyncratic liver toxin diclofenac (see Boelsterli 2003) dosed at 10 mg/kg to C57BLl/6J mice where the extent of covalent binding (at 24 h) were ca. 1.65, 3.37 and 1.42 pg/mg for liver, kidney and plasma, respectively, (the equivalent figures for diclofenac in the HRN mouse were 1.92, 1.86 and 7.97 pg/mg) (Pickup et al. 2012).

**Table 2:** Covalent binding of fenclozic acid-related material in organs or plasma of C57BL/6J mice 72 h after a single 10 mg/kg oral dose of [^14^C]-fenclozic acid

Animal	pmol equiv/mg protein		
	Liver	Kidney	Plasma
1	10.70 ± 0.32	31.03 ± 1.20	7.74 ± 0.71
2	23.49 ± 1.58	48.49 ± 2.98	12.85 ± 1.07
3	18.79 ± 1.42	40.54 ± 3.28	8.09 ± 1.11
Mean ± SD	17.66 ± 6.47	40.02 ± 8.74	9.56 ± 2.85

Each sample is the mean ± SD of 3 determinations/animal/tissue

## Metabolite profiles of [^14^C]-fenclozic acid in urine, faeces and bile

**Fig. 3:**
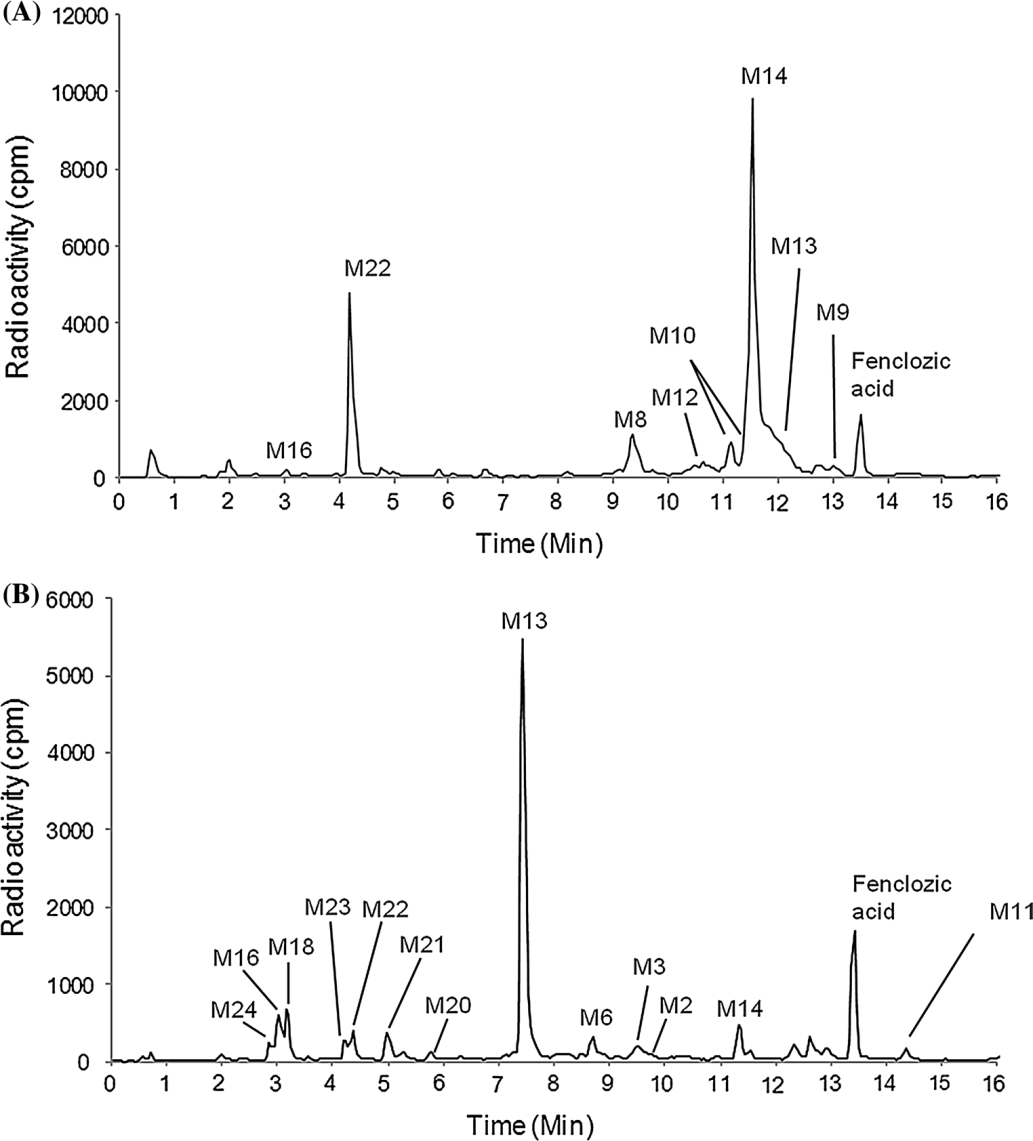
Reconstructed TopCount radiochromatograms of urine **a** and bile **b** following a single 10 mg/kg oral dose of [^14^C]-fenclozic acid to C57BL/6 J mice

Following oral administration of [^14^C]-fenclozic acid at 10 mg/kg to C57BL/6J mice, the majority (ca. 79%) of the radioactivity recovered by 72 h post-dose was present in the urine/cage wash (the radioactivity in the cage wash was presumed to be urinary in origin). UHPLC analysis with radioactivity and mass spectrometric detection revealed the presence of a number of drug-related peaks corresponding to fenclozic acid itself, the acyl glucuronide, glycine and taurine conjugates (**M10, M14** and **M13,** respectively) and an ether glucuronide (**M20**) (Supplementary data Figure S1A). The mass spectra for the acyl conjugates were previously described following studies in HRN mice and the rat (Pickup et al. 2014; Martin et al. 2014). Whilst urine represented the major route for the elimination of [^14^C]-labelled fenclozic acid-related material ca. 21% of the recovered dose was found in the faeces over the 72 h collection period. In contrast to our previous studies on HRN mice, solvent extraction of faeces to recover the excreted fenclozic acid metabolites required relatively aggressive conditions to achieve efficient extractions of radiolabel. The combination of harsh extraction conditions and poor recovery led to concerns that the resulting radio-UHPLC profile (Supplementary data Figure S1B) may be distorted by the degradation of labile metabolites. These concerns led us to undertake a study in bile duct-cannulated mice, which were also sampled for blood plasma and urine as well as bile at 6 h post-dose. Urine, bile and plasma were profiled using UHPLC analysis with radioactivity and mass spectrometric detection (representative reconstructed TopCount radiochromatograms for urine and bile are given in Fig. 3a, b, respectively). Analysis of plasma revealed that the major circulating component was [^14^C]-fenclozic acid itself, with traces of the glycine conjugate also detected (Supplementary data Figure S2). As illustrated in Fig. 3, the urinary and biliary profiles were composed of unchanged fenclozic acid together with a range of oxidised and conjugated (both ether and acyl) metabolites. The profiles of both urine and bile were, however, dominated by acyl conjugates. For urine from bile duct-cannulated mice, as seen in the non-cannulated animals (Supplementary Figure S1A), the acyl glycine conjugate (**M14**) was the most abundant accounting for ca. 40% of the total radioactivity, but acyl taurine (**M13**, 13.3%), glucuronide (**M10**, 4.5%) and glutamyl (**M12**, 2.5%) conjugates were also noted, as well as an ether glucuronide of an oxygenated metabolite (**M22**, 14.7%), which between them accounted for more than 75% of the total radioactivity in the eluent (see Fig. 3a; Table 3). Oxidative decarboxylation, leading to a side-chain shortened metabolite (**M8**, 8.1%) was also observed with its’ corresponding glycine conjugate (**M9**, 1.5%) representing a minor product. Of particular interest, however, was the presence of a trace amount of an oxygenated cysteineglycine metabolite (**M16)**, amounting to ca. 0.6% of the total radioactivity, indicative of reactive metabolite formation. Bile metabolite profiles (Fig. 3b), in contrast to urine, were dominated by the presence of large amounts of the taurine conjugate (**M13**, ca. 43% of total radioactivity) and fenclozic acid (11%). Glycine (**M14**, 3.6%), carnitine (**M11**, 1.2%) and ether glucuronide (**M22**, 2.5%) conjugates were also detected, but the glutamyl, acyl glucuronide and side-chain shortened metabolites seen in urine were absent from bile. The biliary profile, however, provided further evidence of reactive metabolites in the form of a cluster of metabolites formed from the oxygenated glutathione (**M18**, 4.2%) the cysteineglycine (**M24**, 1.9%) and oxygenated cysteineglycine (**M16**, 5.4%) metabolites (Fig. 3b; Table 3) which between them accounted for over 11% of the total radioactivity in the radio chromatogram. Bile duct-cannulated rats administered unlabelled fenclozic acid also showed this type of metabolite and detailed descriptions of the mass spectrometric data for these metabolites are described in detail in this previous work (Martin et al. 2014).

**Table 3:**
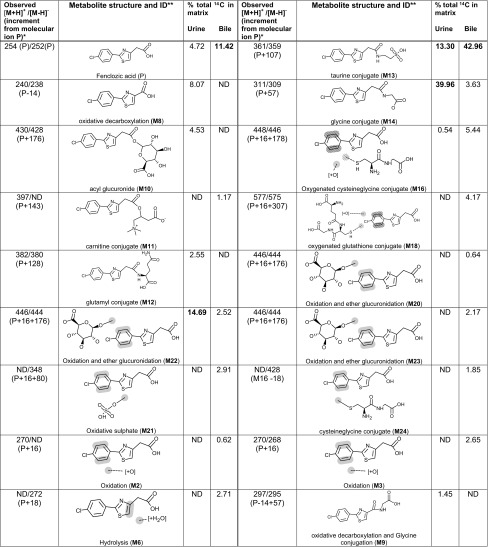
Structures and quantities of [^14^C]-fenclozic acid metabolites in the urine and bile of C57BL/6J mice

Values in bold type denote peak corresponding to >10% total chromatogram radioactivity

Assigned metabolites corresponded to 89.8 and 84.9% of the total radioactivity observed for urine and bile chromatograms, respectively

Highlighted yellow bonds on structures indicate likely sites of metabolism

*ND* Not detected

* Masses correspond to ^12^C isotope ion

** Metabolite nomenclature is in concordance with Martin et al. (2014) where applicable

## Discussion

As indicated in the introduction, despite the best efforts of those involved at the time, the cause of the hepatoxicity observed in human volunteers in clinical trials, but not in any of the pre-clinical species, defied explanation at the time of fenclozic acids’ withdrawal from development (Alcock 1970). More recently, in vitro investigations provided clear evidence of high levels of NADPH-dependant CVB to hepatic microsomes and hepatocytes (Rodrigues et al. 2013) when [^14^C]-fenclozic acid was incubated in the presence of microsomes, but only when supplemented with NADPH (125.8 + 20.1, 70.1 + 19.8 and 38.5 + 6.8 pg equiv./mg protein for human, dog and rat, respectively) and hepatocytes (539.0 + 69.6, 269.7 + 143.5 and 74.9 + 13.6 pg equiv./mg protein for dog, rat and human, respectively). That investigation also demonstrated that CVB to microsomes was reduced in the presence of glutathione or cysteine, suggesting that oxidative metabolism to a soft nucleophile was responsible. However, despite this strong evidence of oxidative metabolism in the in vitro studies, no likely metabolites were detected in incubations with either microsomes or hepatocytes, and ‘trapping’ experiments for reactive metabolites were also negative. In the present study, a histopathological examination detected effects on the liver in mice following a single oral dose of 10 mg/kg. Thus, dosing with [^14^C]-fenclozic acid resulted in acute centrilobular hepatocellular necrosis (Fig. 1) for two out of three animals. However, inflammatory cell infiltration was minimal and was seen only in the areas affected by centrilobular necrosis with no other regions affected. The minimal degree of inflammatory cell infiltration could have been due to the protective anti-inflammatory effects of the drug or it could be that the acuteness of the lesion had not allowed time for inflammation to take place (or indeed a combination of the two). As noted earlier, when fenclozic acid was administered at the same dose as in the present study to HRN mice where hepatic cytochrome P450s were non-functional, there was no evidence of any similar necrosis (Pickup et al. 2014). It is therefore tempting to ascribe this type of effect to P450-related production of toxic metabolites. The present study has shown that CVB to proteins also occurs in vivo with the highest levels seen for kidney followed by liver and plasma. The levels of CVB noted for C57BL/6J mice in the liver 72 h post-dose were 17.66 ± 6.47 pmol equiv/mg protein (whilst for the HRN animals the equivalent figure was 13.43 ± 3.35 pmol equiv/mg protein) (Pickup et al. 2014). As noted earlier these levels of CVB were much higher than those seen for an equivalent dose of the NSAID diclofenac (Sarda et al. 2012) which is a well-known idiosyncratic hepatotoxin in humans (Boelsterli 2003). The lack of metabolites seen in in vitro incubations is in stark contrast to the situation observed in vivo, both during the drugs development (Foulkes 1970) and, more recently in studies undertaken in HRN mouse (Pickup et al. 2014), where extensive conjugation was seen, and the bile duct-cannulated rat (Martin et al. 2014) where biotransformation to both oxidised and conjugated metabolites were detected. The metabolite profiles for fenclozic acid obtained in the present study in C57BL/6J mice also show that the bulk of the metabolites excreted in both urine and bile are formed through direct metabolism of the carboxylic acid moiety to form conjugates, particularly glycine (**M14**) and taurine (**M13**). However, there was also clear evidence of significant oxidative metabolism on the chlorobenzene ring (followed by conjugation to an ether glucuronide) (**M22**), as well as a small amount of oxidative decarboxylation (as previously seen in the mouse for diclofenac (Sarda et al. 2012). Interestingly, as well as in vivo covalent binding to proteins, metabolites detected in the form of the oxygenated cysteineglycine metabolite (**M16**) were seen in both urine and bile with, in addition, the oxygenated glutathione (**M18**) and cysteineglycine (**M24**) metabolites detected in bile, provide further evidence for the formation of reactive metabolites, and their detoxication by glutathione, in the form of soft electrophiles. These glutathione-derived metabolites, particularly those excreted in the bile, between them accounted for a significant portion of the dose (ca. 11%). This relatively large contribution of metabolites linked to reactive metabolite production should perhaps be contrasted with the complete absence of detectable quantities of any downstream metabolites of glutathione conjugates detected in the bile of mice dosed at 10 mg/kg with diclofenac (Sarda et al. 2014). It seems increasingly likely therefore that the toxicity of fenclozic acid observed in humans was, at least in part, the result of the formation of hepatotoxic reactive metabolites as the products of P450-related oxidative metabolism. However, despite the evidence for bioactivation to reactive metabolites indicated in previous studies in the rat (Martin et al. 2014) and in this investigation in the mouse, only limited effects were observed on the livers of the latter here, and no effects were reported in the many pre-clinical toxicology studies performed during the drugs’ development. This might reflect differences in the metabolic flux through the P450-toxication pathway(s) versus the detoxication alternatives provided by conjugation in animals compared to humans exposed to the drug with the proportion of the dose undergoing metabolic activation being much higher in the latter. Such species differences can have profound effects as we have recently shown in the case of acetaminophen where, despite being dosed to the cynomolgus monkey at significant multiples of that needed to cause hepatotoxicity in humans, produced no adverse effects on the liver (Yu et al. 2014). This lack of toxicity is despite the cynomolgus monkey possessing a CYP2E1 enzyme with very high structural homology to that of humans and, based on in vitro results, did show CVB for acetaminophen (Martignoni et al. 2006). However, in vivo, the cynomolgus monkey preparations showed little evidence for quinone imine production, with the majority of the drug apparently metabolised via conjugation (primarily to the glucuronide). A similar explanation, related to the relative flux of fenclozic acid through the various metabolic reactions may also account for differences in DILI seen between humans and the various pre-clinical animal species in which it has been studied.

## Electronic supplementary material

Below is the link to the electronic supplementary material.

Supplementary material 1 (DOCX 101 kb)
