# Supplementary material for: Acute liver effects, disposition and metabolic fate of [14C]-fenclozic acid following oral administration to normal and bile-cannulated male C57BL/6J mice
Source: Arch Toxicol. 2016 Nov 28;91(7):2643–53. doi: 10.1007/s00204-016-1894-5 (PMC5489613; doi:10.1007/s00204-016-1894-5)

**Supplementary Figures: Acute Liver Effects, Disposition and Metabolic fate of [^14^C]-Fenclozic Acid Following Oral Administration to Normal and Bile-Cannulated Male C57BL/6J** **Mice.** Kathryn Pickup et al

**Figure S1.** Typical in-line TopCount radiochromatograms generated from LC/MS of excreta from of C57BL/6J mice following a single 10 mg/kg oral dose of [14C]-fenclozic acid. (A) urine (B) faeces.

**(A)**


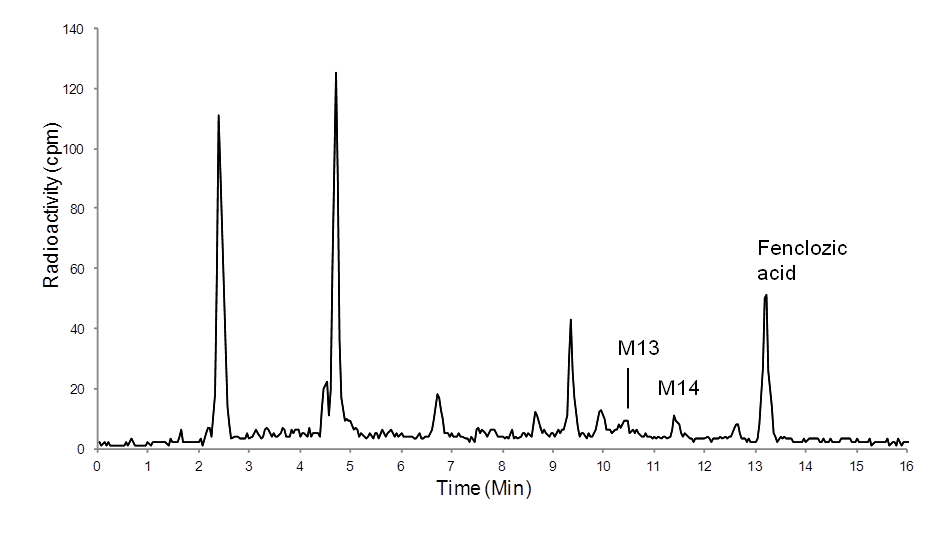


**(B)**


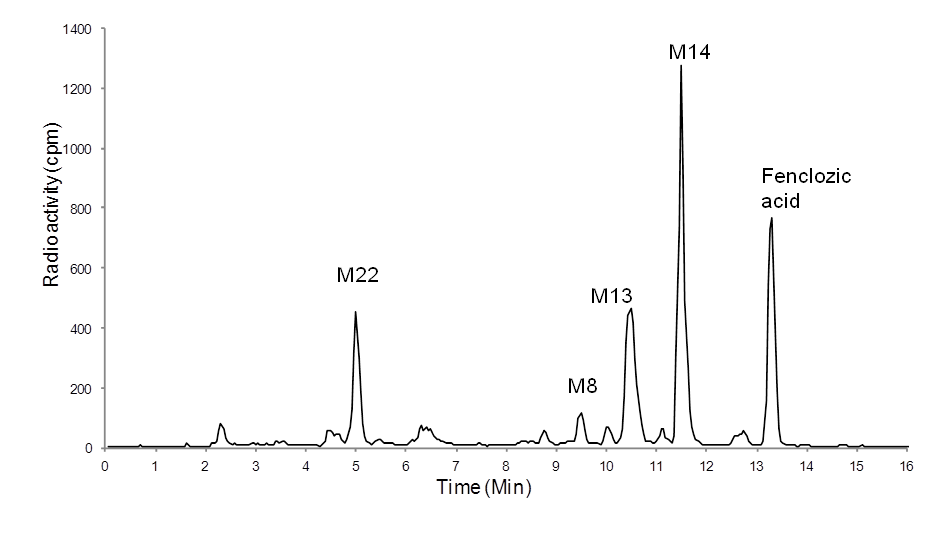


**Figure S2.** TopCount reconstructed radiochromatogram of plasma following a single 10 mg/kg oral dose of [14C]-fenclozic acid to C57BL/6J mice

**Figure S2.**


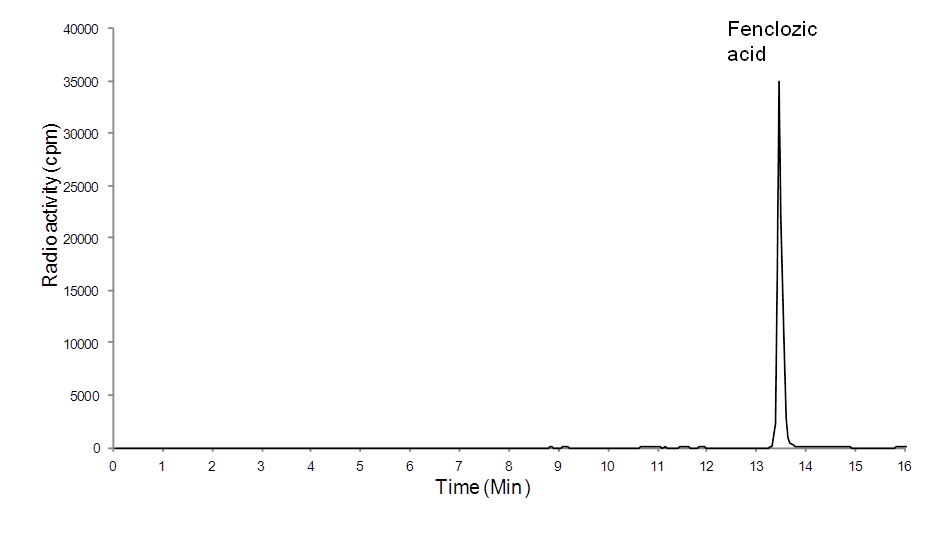

Supplement: Supplementary file 1 — Supplementary material 1 (DOCX 101 kb) [file 204_2016_1894_MOESM1_ESM.docx]
